# Quantitative proteomic analysis of cell envelope preparations under iron starvation stress in *Aeromonas hydrophila*

**DOI:** 10.1186/s12866-016-0769-5

**Published:** 2016-07-22

**Authors:** Zujie Yao, Zhihong Wang, Lina Sun, Wanxin Li, Yan Shi, Ling Lin, Wenxiong Lin, Xiangmin Lin

**Affiliations:** Fujian Provincial Key Laboratory of Agroecological Processing and Safety Monitoring, School of Life Sciences, Fujian Agriculture and Forestry University, Fuzhou, 350002 People’s Republic of China; Key Laboratory of Crop Ecology and Molecular Physiology of Fujian Universities, Fujian Agriculture and Forestry University, Fuzhou, 350002 People’s Republic of China; Agroecological Institute, Fujian Agriculture and Forestry University, Fuzhou, 350002 Fujian People’s Republic of China

**Keywords:** *Aeromonas hydrophila*, Cell envelope, Dimethyl labeling, Iron homeostasis

## Abstract

**Background:**

Iron homeostasis is an essential process over the entire lives of both hosts and bacterial pathogens, and also plays roles in many other metabolic functions. Currently, knowledge is limited on the iron scavenging mechanism of the cell envelope in the aquatic pathogen, *Aeromonas hydrophila*. To understand the iron homeostasis mechanism in *A. hydrophila*, a dimethyl labelling based quantitative proteomics method was used to compare the differential expression of cell envelope proteins under iron starvation.

**Results:**

A total of 542 cell envelope proteins were identified by LC-MS/MS, with 66 down-regulated and 104 up-regulated proteins. Bioinformatics analysis showed that outer membrane siderophores, heme and iron receptors, periplasmic iron binding proteins, inner membrane ABC transporters and H^+^-ATP synthase subunits increased in abundance while iron-cluster proteins, electron transport chain and redox proteins were down-regulated. Further q-PCR validation, in vivo addition of exogenous metabolites, and an enzyme inhibition assay revealed that redox, the energy generation process, and ATP synthase elevated the susceptibility of *A. hydrophila* to iron starvation.

**Conclusions:**

Our study demonstrates that the redox and energy generation process, and ATP synthase in *A. hydrophila* may play critical roles in iron acquisition under conditions of iron-stress. An understanding of the iron scavenging mechanism may be helpful for the development of strategies for preventing and treating *A. hydrophila* infection.

**Electronic supplementary material:**

The online version of this article (doi:10.1186/s12866-016-0769-5) contains supplementary material, which is available to authorized users.

## Background

Iron is the second most abundant metal in the earth’s crust; however, ferrous iron, the useable form, is scarce, due to an oxidative environment [1–3]. It is an essential element for most bacteria with irreplaceable functions in many basic biological processes, including the Fe^3+^ cofactor of metabolic respiratory chain reactions. These include photosynthesis, N_2_ fixation, methanogenesis, respiration, tricarboxylic acid (TCA) cycle, and DNA biosynthesis [2, 4]. To prevent the invasion of bacteria, hosts, including humans, have evolved to minimize the concentration of free iron by secreting transferrin and lactoferrin, which have high affinity for iron [5]. Therefore, bacteria have evolved effective ways to survive in this harsh environment. Under this process, cell envelope proteins in Gram-negative bacteria, including periplasmic, outer and inner membrane proteins, are essential in the transport of iron related compounds into the intracellular environment. The cell envelope proteins related to iron homeostasis mechanisms are well studied in *Escherichia coli*, in which outer membrane proteins (OMPs), such as CirA, FecA, FepA, BtuB, FhuA, FhuE, and YbiL, serve as receptors of siderophores or heme to transport iron into the periplasmic space [6]. Some other OMPs, such as OmpW, TsX and OmpX function as either iron exporters or receivers [7]. During this process, the periplasmic protein TonB and inner membrane proteins (IMPs) ExbB and ExbD form a protein complex to mediate the transport of iron compounds from the outer membrane to periplasm [8]. The periplasmic binding proteins, such as FepB, FhuB, FhuD and FecB in *Escherichia coli* deliver iron compounds to ATP-binding cassette (ABC) transporters in the cytosolic membrane and then into the cytosol [5, 6, 9, 10]. Meanwhile, iron starvation triggers some complex networks and metabolic pathways via the *fur* gene family which regulates iron homeostasis [11].

It is well known that the aquatic pathogen, *Aeromonas hydrophila,* causes serious disease outbreaks in numerous farmed fish populations, and leads to big economic losses in the aquaculture and fishery industry, annually [12–14]. However, knowledge is limited on the iron scavenging mechanism of this pathogen, especially for the role of the cell envelope proteins during the competition for iron resources with the host. In this study, the differential expressions of the envelop proteins *of A. hydrophila* were compared in the presence/absence of the iron chelating medium by a dimethyl labelling based quantitative proteomic method. Bioinformatics analysis found some important biological processes involved. Furthermore, some of the altered proteins were validated by quantitative polymerase chain reaction (q-PCR) analysis and subjected to related functional validation. We provide the first report, to our knowledge, on the iron homeostasis functions of cell envelope proteins in *A. hydrophila*.

## Results

### Characterization and identification of the envelope protein fraction in *A. hydrophila*

**Fig. 1:**
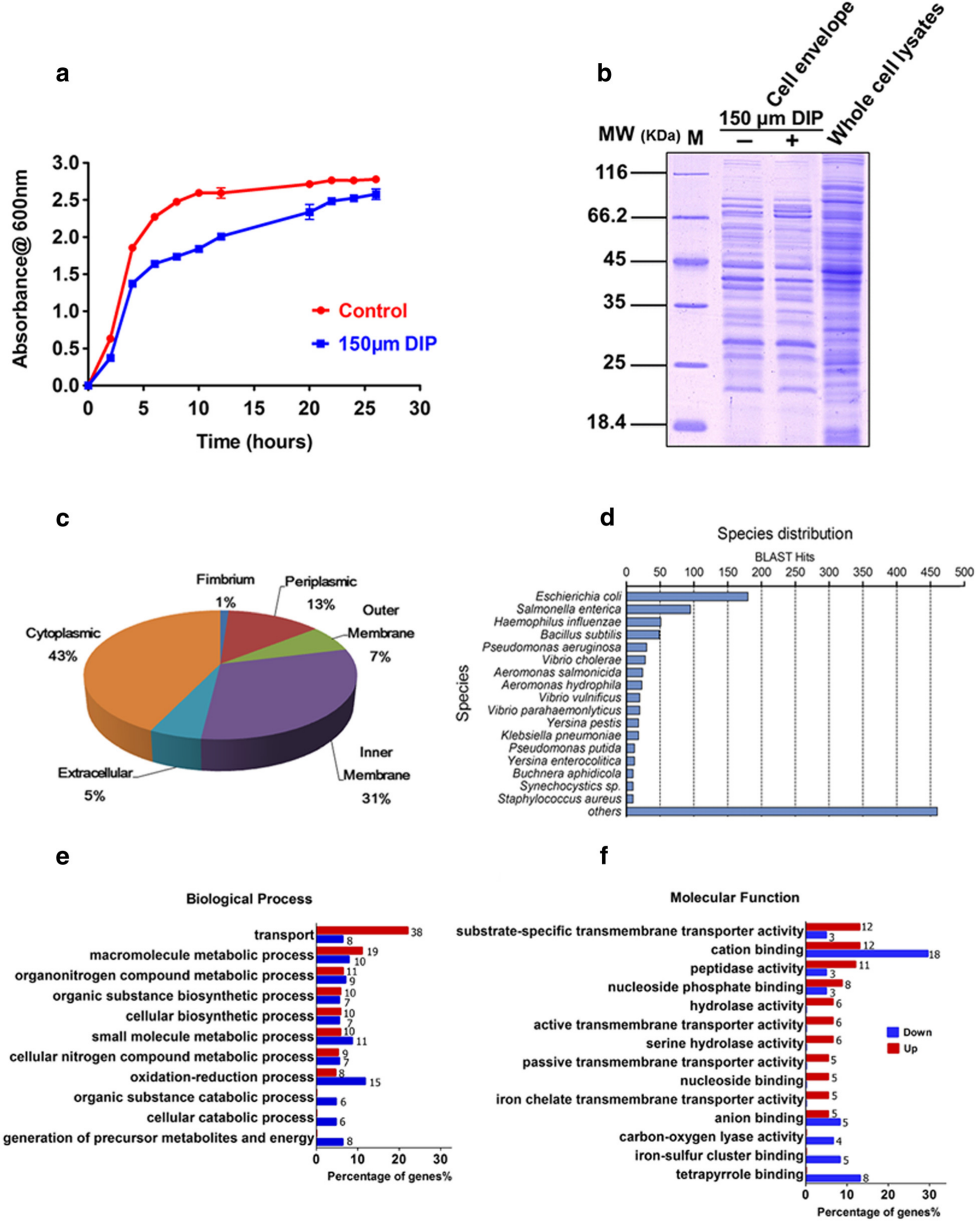
Comparative characteristics of cell envelope protein of *A. hydrophila* in iron limited medium **a** Growth curve of *A. hydrophila* ATCC 7966 with and without 150 μM DIP in LB medium; **b** CBB-stained SDS PAGE map of the cell envelope of *A. hydrophila* with and without 150 μM DIP and whole cell lysates of *A. hydrophila* as the comparison. Lane M contained molecular mass standards; **c** The subcellular localization of the identified proteins from MS results predicted by online software Gneg-mPLoc; **d** Blastp top-hit species distribution using local Blast2GO. Numbers of top hit sequences from Blastp were calculated for each species; **e** and **f** Gene ontology categories for the differentially expressed proteins of *A. hydrophila* in iron starvation using local Blast2GO analysis and classified into biological processes (**e**) and molecular functions (**f**). The red and blue bars indicate up-regulation (*red*) and down-regulation (*blue*) differential ratios of genes, respectively. Each related gene numbers are showed on the right of bars

According to the growth curve of *A. hydrophila*, the strain treated with 150 μM 2, 2′-dipyridyl (DIP) grew slower than the control strain, especially after the middle exponential phase (Fig. 1a). To investigate the biological functions of envelope proteins in iron homeostasis, bacterial envelope proteins in presence/absence of DIP with the concentration of 150 μM were extracted. SDS-PAGE separation showed some significant differences between DIP treatment and the control, and the whole cell lysates of *A. hydrophila* were made to probe the cell envelope preparation enrichment in the preparations (Fig. 1b). After comparing with these differences between DIP treatment and non-treatment, the extracted cell envelope proteins of *A. hydrophila* were then in-solution digested and labeled by Stable Isotope Dimethyl. Further LC MS/MS analysis identified 837 proteins with at least two peptides required for identification and a false discovery rate (FDR) less than 1 % filtered in the total number of 1024 proteins (Table 1 and a complete list in Additional file 1: Table S1).

**Table 1:** Selected identification of significantly differentially expressed proteins of *A. hydrophila* ATCC 7966 under iron stress using dimethyl labeling quantitative proteomics

Accession Name	Description	Match peptides	Coverage %	MW	Ratio	Location Prediction
K1JDT3_AERHY	Uncharacterized protein TonB-dependent	13	56.4	33.65	45.53	PM.
K1JLD4_AERHY	hemoglobin/transferrin/lactoferrin receptor family protein	21	33.4	77.04	40.78	OM.
R4VT74_AERHY	Proprotein convertase P-domain-containing protein	24	35.4	88.48	28.78	Ex.
A0KJP9_AERHH	TonB-dependent siderophore receptor	39	61.8	71.66	23.97	OM.
R4VC61_AERHY	Hemin receptor	30	52.8	80.03	22.83	OM.
R4VMU5_AERHY	Uncharacterized protein	13	44	49.37	22.67	Ex.
D2XPP9_AERHY	Hemolysin	30	53.3	68.78	22.46	Ex.
R4W1J6_AERHY	Methyl-accepting chemotaxis protein	2	2.4	72.99	22.26	IM.
R4VA05_AERHY	Outer-membrane heme receptor	39	66.9	76.73	19.06	OM.
A0KIU6_AERHH	Proprotein convertase P-domain	21	31.8	88.41	15.73	Ex.
R4VVH5_AERHY	Uncharacterized protein	6	76.1	8.64	0.16	IM.
A0KL15_AERHH	Bordetella uptake gene family protein	12	44.9	34.03	0.16	IM.
R4VV91_AERHY	EcoEI R domain-containing protein	2	2.7	85.41	0.15	IM.
R4V7Y6_AERHY	Cytochrome c4	9	37.6	21.29	0.15	PM.
R4VH33_AERHY	Cytochrome c551 peroxidase	9	28	35.62	0.15	PM.
A0KLX1_AERHH	DeCa-heme c-type cytochrome	15	24.5	79.84	0.14	CM. Ex. PM.
R4VKJ3_AERHY	Uncharacterized protein	2	14.6	17.35	0.13	IM.
A0KL28_AERHH	Cytochrome c-type protein	3	13.7	22.14	0.11	PM.
K1J5J8_AERHY	Uncharacterized protein	19	26.9	72.17	0.11	OM.
R4VF53_AERHY	Cytochrome c553	10	66	15.31	0.09	PM.

Note: PM., OM., IM., Ex. and CM. mean the location of periplasm, outer membrane, inner membrane, extracell and cytoplasm, respectively

According to the online location prediction software, we found that these identified proteins included 447 cytoplasmic proteins (account for 43 %), 323 inner membrane proteins (31 %), 138 periplasmic proteins (13 %), 71 outer membrane proteins (7 %), 55 extracellular proteins (5 %), and 12 fimbrium proteins (1 %) with some overlapping proteins in the predicted locations (Fig. 1c).

### Differential expression of envelope proteins in *A. hydrophila* in iron-limited medium

To further investigate the biological behavior of *A. hydrophila* under iron-limiting conditions, dimethyl labeling based quantitative proteomic technology was used to analyze the differential expression of envelope proteins. In the 837 identified proteins, 170 membrane envelope proteins were found to be differentially expressed. Taking overlapping locations into account, 104 proteins including 37 OMPs, 50 IMPs, 25 periplasmic proteins and three fimbrial proteins were up-regulated, while 66 proteins including seven OMPs, 39 IMPs and 28 periplasmic proteins decreased in abundance in the iron starvation medium.

Of these altered proteins, seven outer membrane proteins, A0KJP9, R4W0J5, R4VC61, A0KJN3, A0KGW8, R4VA05, and K1JLD4, which serve as ferrienterobactin, ferrichrome, hemin, siderophore and heme receptors, and at less five periplasmic ABC transporters (R4VG84, R4VTC6, R4VQ44, A0KG07, and R4VTR3) and ExbB family protein (R4W2T6) increased in abundance. However, OmpW (R4VIJ9) of *A. hydrophila* presented a down-regulated trend, although its biological function is still unknown. Interestingly, besides the outer membrane siderophore receptors, there were more outer membrane proteins altered, including R4VAF7 (OmpK family, up), A0KK67 (FadL family, up), A0KLX3 (outer membrane translocase, down), R4VCH3 (OmpD family, up), R4VRX0 (major outer membrane lipoprotein, up), A0KNY2 (ABC-type efflux system secretin component, up), R4VNF2 (OmpA family lipoprotein, up), A0KHF6 (Maltoporin, down) and R4VR45 (Chitoporin, up).

Meanwhile, seven c-type cytochromes (A0KQV7, R4VNL8, R4VQS4, R4V7Y6, R4VH33, A0KL28, and R4VF53) decreased in abundance. These proteins contained iron ion binding domains suggesting they reduce the use of iron and lead to the decrease of the respiratory electron transfer chain under iron starvation. On the contrary, ATP synthase subunits (R4VLV7, R4W2U5, and R4VJ80), the final terminal point of cellular respiration, were found to be up-regulated. While the purpose of this opposite phenomenon is unknown, we hypothesize that it provides an ATP source for ferrous iron scavenging.

Proteomics analysis also showed that almost all of the altered proteins located in the extracellular space were up-regulated, such as hemolysin (R4VSQ0 and D2XPP9) and fimbrial protein (R4VSB4) could be explained as an attempt by the cell to strengthen the acquisition of scarce iron ions from outside and try to escape from the threatening surroundings. In addition, many proteases such as Q49KA8 (Serine protease), A0KIU6 (Proprotein convertase P-domain), A0KGX4 (Putative metalloprotease), R4VU01 (Hg (II) reductase) and A0KFK2 (glycosidase) were found to increase in extracellular space.

### Investigation of the classification and functional annotation analysis of altered proteins

To further characterize the altered envelope proteins, a biological functional classification was clustered using bioinformatic analysis. The local Blast2GO software was chosen to maximize homologous gene/protein annotations because of the limitation of Gene Ontology (GO) annotations of *A. hydrophila*. The BLASTP program was run against the Swiss-Prot database, producing 131 (74.0 %) matches from a total of 170 candidates with blast expected values ≤10^−3^ using BLAST2GO. Figure 1d shows the Basic local alignment search tool (BLAST) hit distribution across various species of organisms for all altered sequences in the dataset. Further analysis identified the most enriched GO terms comparing the up- and down-regulated proteins clusters including transport (22 % Go terms up-regulated and 6.3 % down-regulated), macromolecule metabolic process (11 % up, 7.8 % down), oxidation reduction process (4.6 % up, 11.7 % down), small molecule metabolic process (5.8 % up, 8.6 % down), generation of precursor metabolites and energy (6.3 % down) in biological process category and cation binding (13 % up, 29.5 % down), tetrapyrrole binding (13 % down), iron-sulfur cluster binding (8.2 % down), active transmembrane transporter activity (6.5 % up) and iron chelating transmembrane transporter activity (5.4 % up) in molecular function category (Fig. 1e and f).

### Validation of selected altered proteins at the mRNA level using q-PCR

**Fig. 2:**
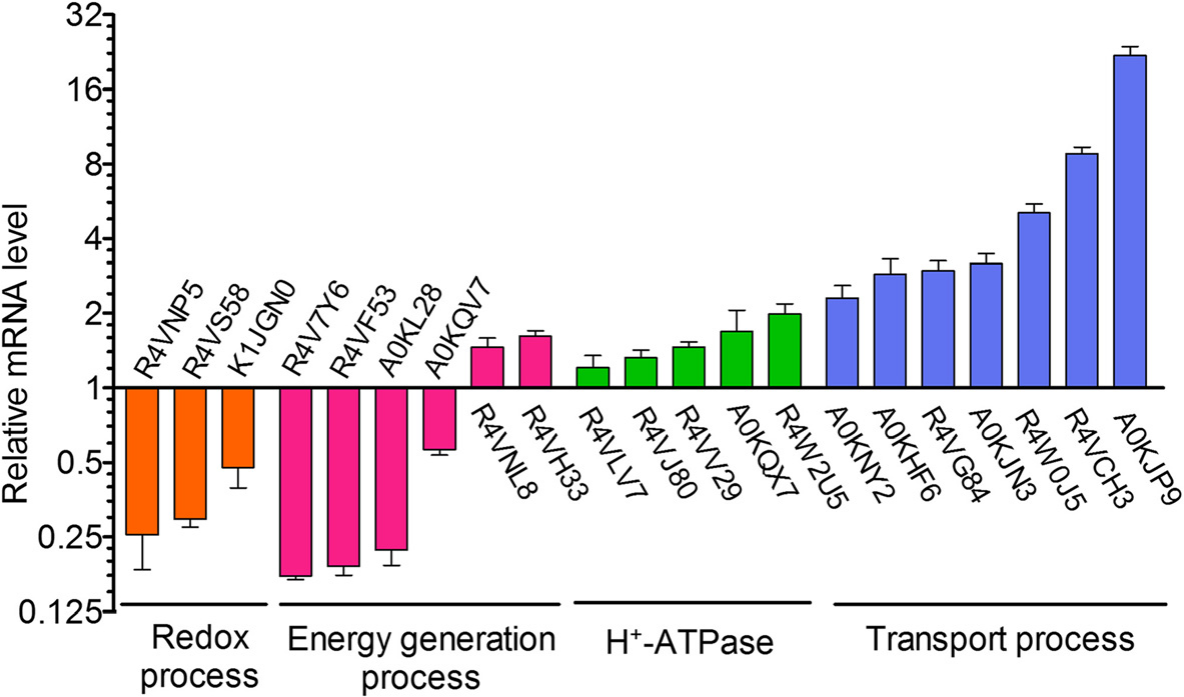
The validation of relative expression behaviors of transport, redox, and energy generation processes were selected and verified by q-PCR The mRNA level of 12 transport related genes which were up-regulated at the protein level (*blue*), 6 oxidation-reduction and 3 energy generation process related genes which were down-regulated at the protein level (*red*), were analyzed by q-PCR

According to the proteomic results and data analysis, up-regulated transport processes, down-regulated redox and energy generation process were the major response characteristics of envelope proteins in iron starvation. To confirm this, we investigated the mRNA levels of a total of 21 selected genes found to be altered in the proteomic analysis, using a q-PCR method. Of these 21 genes, five genes (R4VLV7, R4VV29, R4W2U5, R4VJ80, A0KQX7) were H^+^-ATPase subunits, seven genes (A0KNY2, A0KHF6, R4VG84, A0KJN3, R4W0J5, R4VCH3, A0KJP9) were related with the transport process, six genes (R4V7Y6, R4VF53, A0KL28, A0KQV7, R4VNL8, R4VH33) were involved in the redox process and three genes (R4VNP5, R4VS58 and K1JGN0) were involved in the energy generation process. Results showed that most of the transcription levels of these 21 related genes were consistent with our proteomics conclusions (Fig. 2). In the selected transport-process related genes, the mRNA level of A0KJP9, a TonB-dependent siderophore receptor, displayed a very significant up-regulation (21.91 folds) under iron starvation, suggesting its crucial role in iron uptake. On the other side, the transcription of other candidate genes increased from 1.5 to 8.8 fold, except for R4VLV7 and R4VJ80, which only increased slightly, with a ratio of 1.2 and 1.3, respectively.

In the redox process and energy generation process, the down-regulated mRNA levels of R4V7Y6, R4VNP5, R4VF53, A0KL28, and R4VS58 were in agreement with protein levels, while K1JGN0, A0KQV7 only changed slightly. Interestingly, the transcription of R4VNL8 (ammonia-forming cytochrome c nitrite reductase) and R4VH33 (cytochrome c551 peroxidase) increased, contradictory to the proteomic results. In summary, up to 81 % (17/21) of genes displayed the same directional change in both protein and mRNA levels, 19 % (4/21) showed no correlation, while only 10 % (2/21) were dysregulated in the opposite direction.

### TCA related exogenous metabolites elevate susceptibility of *A. hydrophila* to iron starvation

**Fig. 3:**
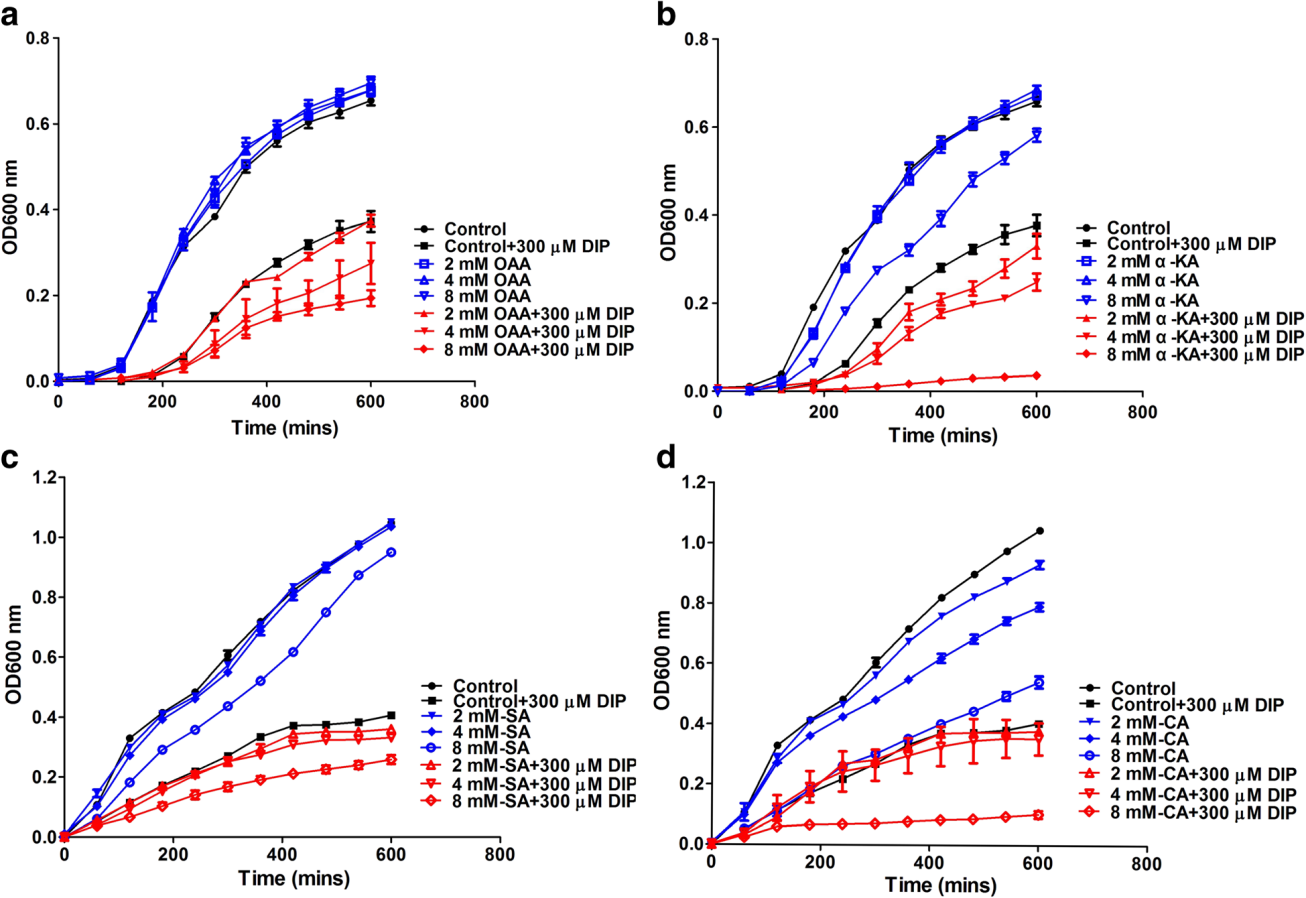
Effect of exogenous metabolites on susceptibility of *A. hydrophila* to iron starvation. **a**–**d** The growth curve of *A. hydrophila s*train in 300 μM DIP mM oxaloacetic acid (OAA), α-ketoglutarate (α-KA), succinic acid (SA) and citric acid (CA) treatment, respectively. The concentration of these four reagents are in the gradient of 0, 2, 4 and 8 mM. The growth kinetics of treated cells were recorded by absorbance measurements at OD600 nm, at 30 °C with the Multi-mode detection platform

In this study, we found the down-regulation of the TCA related energy generation processes under iron starvation, using a proteomics approach. We assume that the down-regulation of the energy generation process takes part in the iron homeostasis mechanism rather than simply stimulation from iron depletion. To validate this, serial dilutions of the TCA related exogenous metabolites were added in LB medium with or without DIP to stimulate the TCA process. As shown in Fig. 3, low of concentration exogenous metabolites alone did not affect bacterial growth while high levels of metabolites, such as Oxaloacetic acid (OAA), α-Ketoglutarate (α-KA), Succinic acid (SA) and Citric acid (CA), did depress the bacterial growth rate. However, when the exogenous metabolites were added in LB medium with 300 μM DIP, most of them elevated susceptibility of *A. hydrophila* to iron starvation, especially α-KA and CA, which both depressed bacterial growth significantly at 8 mM (Fig. 3b and d).

### H^+^-ATP synthase activity was increased in the iron starvation environment

**Fig. 4:**
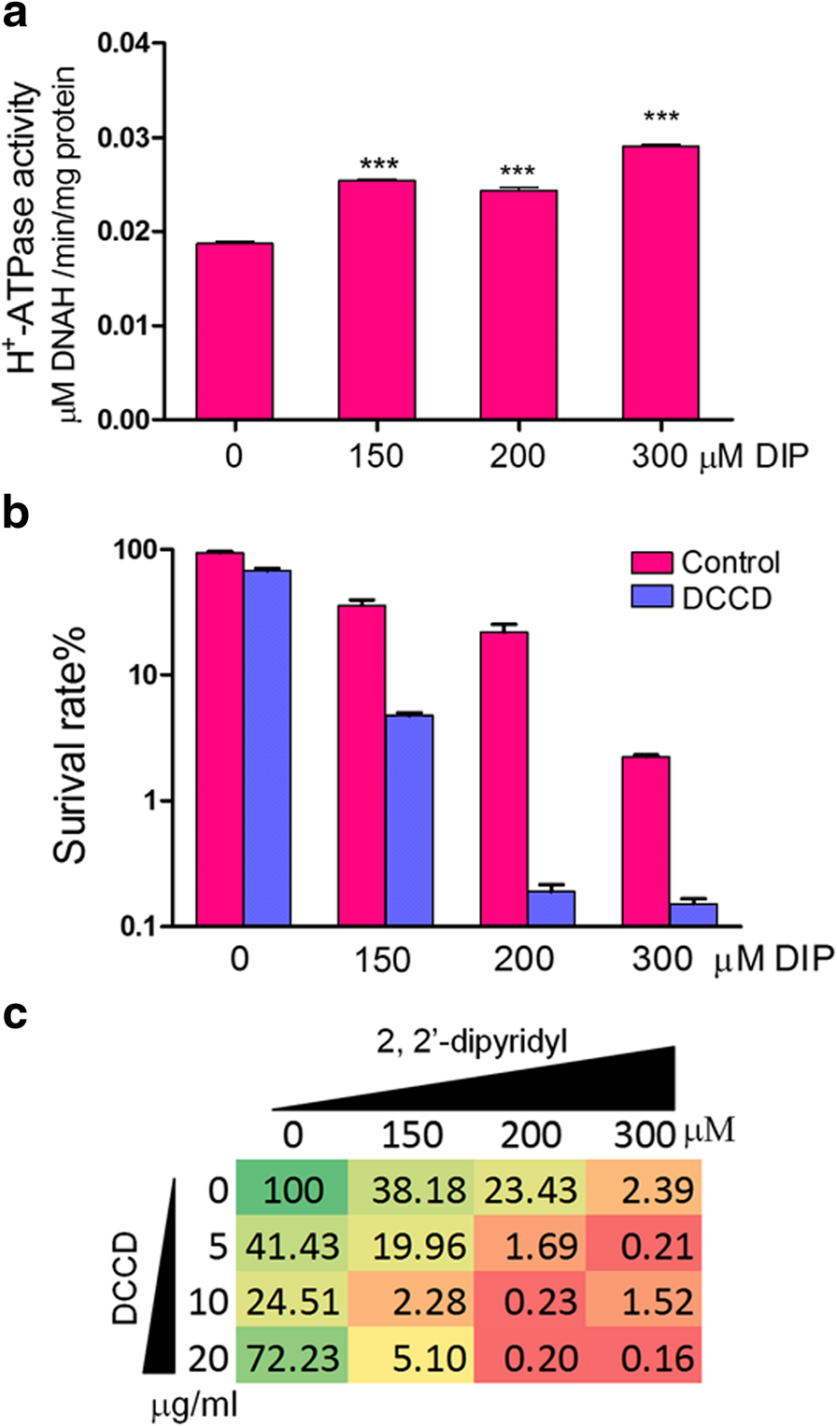
The functional validation of ATP synthase under iron starvation conditions. **a** The H^+^-ATP synthase activity of *A. hydrophila* in a serial of DIP concentrations in 0, 150, 200 and 300 μM were measured spectrophotometrically at 340 nm using the H^+^-ATPase assay kit **b** The survival ratio of *A. hydrophila* when treated with 20 μg/mL DCCD in a serial dilution of DIP concentrations in 0, 150, 200 and 300 μM for 12 h and observed by colony counting, respectively. **c** Color grading of the effect of a serial of concentrations of DIP from 0 μM to 300 μM and DCCD from 0 μg/mL to 40 μg/mL. The colony amounts of bacteria without treatment were normalized to 100 as control

We further confirmed the behavior of H^+^-ATP synthase under iron limited conditions. H^+^-ATPase activity was measured with a serial dilution of DIP treatment. Compared to control, the H^+^-ATPase activities of *A. hydrophila* were increased in response to iron starvation (Fig. 4a), which are consistent with our proteomics results. We then investigated the biological characteristics of H^+^-ATP synthase in iron starvation using N, N’-Dicyclohexylcarbodiimide (DCCD), an inhibitor of ATP synthase. When treated with 20 μg/mL DCCD, the bacterial survival rate was significantly decreased by 7.5, 117.2 and 14.9 fold with the increase of DIP concentrations 150 μM, 200 μM and 300 μM, respectively (Fig. 4b). To further investigate the extent of functional differences under iron-limitation, an orthogonal test was performed by using serials of DIP and DCCD concentrations and then displayed as color grading according to their survival colony accounting (Fig. 4c). Results showed that, with the increasing of DIP plus DCCD treatments, the survival rates of *A. hydrophila*, were dramatically decreased.

## Discussion

Aquatic pathogenic bacteria *A. hydrophila* cause a fulminant epidemic in aquaculture, including some extremely severe bacterial diseases, such as hemolytic ascitic disease, bacterial septicemia, and fulminant hemorrhage [15, 16]. So far, the response mechanism of *A. hydrophila* under some metal ion deficient conditions is not clear. Other bacteria, in order to respond to physiological iron-deficient conditions, have evolved a series of strategies to compete with the host for the uptake of life-essential iron [17–19]. During this process, the cell envelope has an important effect on the extracellular and intrinsic communications due to its specific position, especially for the competition of essential nutrients in harsh environments [20, 21].

In order to systematically study the iron acquisition mechanism of the bacterial cell envelope system under the external pressure of iron limiting conditions, a dimethyl labeling based quantitative proteomic strategy was used to compare the differential expression of cell envelope proteins in *A. hydrophila*. About 57 % of cell envelope proteins were extracted and identified in the current study. Based on the fact that cytoplasmic proteins contributed to the major contamination in all reported extraction methods, the TSE (Buffer ingredients contain 0.2 M Tris–HCl, pH = 8.0, 0.5 M sucrose, 1 mM Na_2_EDTA) extraction used in this study was documented to produce the cleanest extraction of envelope proteins up to date [22]. Compared to the 52 % enrichment ratio in *E. coli*, using the same method in the mentioned reference, the 57 % of cell envelope proteins enriched in *A. hydrophila*, should be acceptable [21]. Thus, in this study we acquired an acceptable cell envelope protein enrichment in *A. hydrophila*.

In the present study, according to the final quantitative proteomic analysis, which can be summarized into the following statements: Firstly, the expression of iron source receptors, which are located in the bacterial outer membrane, induced iron scavenging. These are high-affinity iron chelators and competitively bind iron from iron sources. Actually, under iron deprivation, there are many reports about the upregulation of proteins involved in regular channel forming proteins, such as siderophore receptor channels, CirA, FecA, FepA, FhuA, FhuE, and YbiL in *E coli* [3], and other channel proteins, such as OmpX in *E.coli* [7]. Those results demonstrated the competitive uptake system of iron in *A.hydrophila* under iron-limiting conditions.

Secondly, after the ferri-siderophores and other iron sources specifically bind to outer membrane receptors, an energy generation system for transport is required. Because there was no ATP energy supply in the outer membrane, the ExbB–ExbD–TonB system assisted ferri-siderophores translocation from the outer membrane to the periplasm in *A. hydrophila* as well as in other bacteria [23–27]. Generally, it is provided the energy by the energy-transduction TonB-ExbB-ExbD protein complex, such like in *E.coli* [28]. Although we could not identify TonB and ExbD proteins in the current study, an ExbB family protein, R4W2T6 was up-regulated three fold, suggesting that TonB-ExbB-ExbD might participate in iron protein uptake in *A. hydrophila* as well. Besides, periplasmic iron binding proteins, such as the heme- and vitamin B12- binding proteins, and the associated cytoplasmic membrane ABC permease transporters mediated the transport of ferri-siderophore complexes across the periplasm and cytoplasm similar to the other fish pathogenic bacteria *Edwardsiella ictaluri* [29].

Thirdly, besides the siderophore receptors are located in outer membrane, there were many OMPs differentially expressed under iron starvation. In our previous study, we found that the outer membrane protein R4VIJ9 (OmpW) was down-regulated in *E.coli* in iron-limited medium [7]. Some other channel proteins were in the identical tread of down-regulated, such as and A0KLX3 (MtrB/PioB family decaheme), and also many found up-regulated channel proteins, such as R4VRK9 (sucrose porin), A0KMI4 (porin), R4VNF2 (OmpA family protein), Q4ZHS9 (major outer membrane protein) and A0KNY2 (outer membrane efflux protein) in iron starvation in this study as well. The biological functions related to iron depletion are largely unknown for these proteins. According to the fact that most of these proteins are the channels on outer membrane and control the influx or efflux of small molecular substrates, they might have an important effect on the controlling and monitoring of the cellular free iron concentration [30, 31]. Because of the channel characteristics of OMPs, the down-regulation of OMPs might influence the influx of iron related substrates, whereas the up-regulation of OMPs might affect the efflux function [32, 33]. Additionally, the peptidoglycan-associated lipoprotein, Pal, and periplasmic TolB increased under iron starvation. The Tol–Pal system was energized by the proton motive force and contributed to maintain the cell envelope integrity [34]. Since Tol–Pal system related proteins are homologous with the TonB-ExbB-ExbD system, they may participate in the iron uptake for bacteria survival [35].

Fourthly, bacteria have to reduce their intracellular redox reaction rates because many functional related enzymes are iron-cluster proteins. Thus, the energy generation system and electron respiration chain were depressed under iron starvation. We assume that the down-regulation of the energy generation process takes part in the iron homeostasis mechanism rather than simply stimulation from iron depletion. The down-regulation of TCA cycle and its related proteins under iron starvation was reported in other bacteria species, such as FrdA, FrdB, FrdC and FrdC in *E. coli* [3], SdhA, CitG, CitB, CitC and PckA in *Staphylococcus aureus* [36]. Smaldone et al., proposed the derepression of *Fur*-regulated FsrA may repress expression of glutamate synthase (GltAB), and finally down-regulate TCA cycle intermediate α-ketoglutarate, and DctP which imports succinate and fumarate [37]. Thus, the down-regulation of TCA cycle under iron-deficient condition demonstrated that the stimulation of the TCA process depresses bacterial growth in iron starvation environment and plays an important role in iron homeostasis.

Finally, the reduction of the electron respiration chain, to some extent, might relieve the danger of bacterial reactive oxygen species (ROS) and help for survival [2, 38, 39]. On the other hand, cells require iron resources such as siderophores or heme urgently for iron homeostasis by transporting iron compounds from the periplasm to cytosol and this process consumes ATP [40, 41]. During this process, ATPase should be activated for ATP support. In this study, the down-regulation of the TCA cycle, cellular respiration and the increasing of ATPase were identified by proteomics and validated in mRNA level by q-PCR. This outcome was consistent with our proteomics result confirms the previous report that mRNA and protein levels do not correlate perfectly and suggests that protein post-translational modifications [42]. The intrinsic reason of this phenomenon remains largely unknown. To our understanding, ATPase subunits do not have iron-sulfur clusters and would not affect the use of limited iron resource [43]. The increasing expression of ATPase may be a compensation mechanism to produce ATP resource for ABC transport as much as possible in the environment where the proton motive force (PMF) is resulting in a decrease. To confirm this hypothesis, we investigated the ATP synthase function under iron limiting conditions using an enzyme activity assay and ATP synthase inhibitor. Our results validated that the activity of H^+^-ATP synthase was activated in iron starvation and the inhibition of its activity would increase the susceptibility of *A. hydrophila* to iron starvation. According to the proteomic results of cell envelope proteins involved in iron starvation, the scheme of iron uptake systems in *A. hydrophila* is represented graphically in the Additional file 2: Figure S1.

## Conclusions

The aim of this study was to investigate the iron homeostasis mechanism in the aquatic pathogen, *A. hydrophila*. To our knowledge, this is the first report on the iron homeostasis functions of cell envelope proteins in *A. hydrophila* by using a dimethyl labeling based quantitative proteomic method. The transmembrane transporter activity and ATP synthase increased significantly in abundance to enhance iron transport and maintain cellular iron homeostasis, while redox processes, precursor metabolites, energy generation processes and other iron-sulfur cluster binding processes decreased significantly in abundance to alleviate the pressure of the iron-limitation, by reducing iron use. In general, this mechanism plays a crucial role in the signal transmission and regulation of the pathogenic bacteria under metal ion limited conditions. A deep understanding of the iron acquisition mechanism of bacteria in the metal ion deficient conditions provided a theoretical support for the invasion mechanism of *A. hydrophila* disease.

## Methods

### Cultivation of bacterial strains

In this study, *A. hydrophila* (ATCC7966) was kindly provided by Prof. Jijuan Cao from Liaoning Entry-Exit Inspection and Quarantine Bureau, Dalian, PR. China. This strain was grown in Luria-Bertani (LB) medium at 30 °C. Iron limitation was conducted by the inclusion of a divalent metal chelator, the ferrous iron binding agent 2, 2′-dipyridyl (DIP) (Sinopharm Chemical Reagent Co., Ltd, Shanghai, China) [44, 45], at 0 or 150 μM in LB medium, respectively. The overnight culture was diluted 1:100 into fresh LB medium with 150 μM DIP at 30 °C with shaking at 200 rpm to late exponential phase (OD600 ~ 1.0).

### Cell envelope extraction

The proteins from the *A. hydrophila* cell envelopes were extracted according to a modified osmotic shock method [22]. Briefly, cells were harvested at late exponential phase by centrifugation at 3,000 x g for 20 min at 4 °C. TSE buffer (0.2 M Tris–HCl, pH = 8.0, 0.5 M sucrose, 1 mM Na_2_EDTA) was subsequently added to the pellet to extract cell envelope proteins by causing the cells to shrink and release their contents. After incubating on ice for 20 min and additional 20 min in the same volume of ice-cold water, the soluble envelope proteins were separated by centrifugation at 16,000 x g for 30 min at 4 °C. Supernatants were concentrated with Amicon® Ultra-4 centrifugal filter devices with 3 K Da cut offs (Millipore), and then frozen at −80 °C before use.

### In-solution digestion and stable isotope dimethyl labeling

The total cell envelope protein concentration was determined using a Bradford assay. Proteins from each sample (100 μg) were reduced with a solution of 200 mM Tris (2-carboxyethyl) phosphine (TCEP) at room temperature for one hour, and alkylated with 25 mM Iodoacetamide (IAA) at room temperature in the dark for 45 min as described previously [46]. Treated samples were precipitated by adding six volumes of ice-cold acetone and incubated at -20 °C for at least 12 h. Then, the samples were centrifuged at 8,000 × g for 10 min at 4 °C, and 100 μL 200 mM Triethylammonium bicarbonate (TEAB) was added for dissolution [47]. Finally, each sample was digested with 5 μg Trypsin protease (Thermo Scientific) at a 1:20 ratio and incubated at 37 °C for 12–16 h. About 25 μg digested peptide from each group was taken out for further stable isotope dimethyl labeling, as described previously [48]. The labeling scheme was: *A. hydrophila* in LB medium with and without 150 μM DIP as the heavy and light isotopes, respectively.

### LC MS/MS analysis for Q-Exactive

Digested and labeled peptides were analyzed with a Q-Exactive mass spectrometer coupled to an Easy-nLC 1000 HPLC instrument (Thermo Fisher Scientific, Bremen, Germany). The peptide mixture was loaded onto an Easy-spray column (C18, 2 μm, 100 Å, 75 μm × 50 cm) and separated with a linear gradient of 3–90 % buffer B (0.1 % Formic acid in acetonitrile) at a flow rate of 250 nL/min over 180 min. Normalized high-energy collision dissociation (HCD) was performed, with the collision energy set at 30 %. The instrument was operated in positive ion mode with an ion spray voltage of 2.3 kV, using a data-dependent top 20 method dynamically choosing the most abundant precursor ions. Isolation of parent precursors was performed with a 300–1800 m/z range and MS/MS was acquired starting at 100 m/z [49, 50].

Fragmentation data were interpreted by Proteome Discoverer version 1.4 against the *Aeromonas hydrophila* database (download from Uniprot database on Dec.1st. 2014) with the search parameters including alkylated cysteine residue by iodoacetamide, trypsin digestion. Protein quantitation and confidence of the dimethyl labeling quantitative method were performed and evaluated using a highly conservative threshold (folds change ≥ 2.0 or ≤ 0.5, FDR < 1 %). The identified proteins with at least two peptides matched and normalized with related peptides in the control were considered for further analysis. Each experiment was performed in at least triplicates and identified in three technical replicate experiments.

### Bioinformatics analysis

The Gene Ontology (GO) terms of the differential proteins in this study were analyzed to characterize the related biological functions. To overcome the disadvantage of poor GO annotations in the *A. hydrophila* database, Blast2GO version 2.7.2 (http://www.blast2go.com/) was used, which is a homology-based approach for gene ontology enrichment analysis [51]. The protein subcellular locations were identified by the program Gneg-mPLoc version 2.0 (http://www.csbio.sjtu.edu.cn/bioinf/Gneg-multi/#) which combines the gene ontology information and functional domain with the sequential evolution information for Gram-negative bacterial proteins [52].

### Validation of proteomic analysis

To determine the mRNA expression, total RNA was isolated from cultured bacterial cells using a TaKaRa MiniBEST Universal RNA Extraction Kit (Takara Shuzo, Otsu, Japan) according to the manufacturer’s instructions. Isolated RNA samples were eluted in 30 μL RNase-free water and the cDNA was generated from 1 μg total RNA using a PrimeScript ™ RT reagent Kit with gDNA Eraser (Takara Shuzo, Otsu, Japan) after calculations to perform one round of the reverse-transcription reaction. The expression of Glyceraldehyde-3-phosphate dehydrogenase (*GAP-1*) was detected as the internal endogenous control, and all samples were normalized to *GAP-1* according to a previously described method (Table 2) [53].

**Table 2:** Sequences of the primer pairs used in this study for q-PCR

Name	Description	Primer	Sequence (5′➔3′)	Nucleotide position	Product size (bp)	Reference
A0KNY2	Outer membrane efflux protein	F	AGTTCGGTCAAGATGCTGTGG	193	193	This study
		R	CGATCTCCTGCTTGGTCTGC	385		This study
A0KQX7	ATP synthase epsilon chain	F	CTCCGTTGCTGACTGCCATC	116	166	This study
		R	GCTTTCGCCTCATCCAAATCG	281		This study
R4VLV7	ATP synthase gamma chain	F	AGGCTTACGACAACGGTGAG	473	199	This study
		R	TAGCGAATCAGCAGGGTATCC	671		This study
R4VV29	ATP synthase subunit alpha	F	CAACGCCGAGTATGTAGAGAAG	912	164	This study
		R	GCTGTGAGGTCAGGAAGATC	1,075		This study
R4W2U5	ATP synthase subunit b	F	ATTGCTGACGGTCTCTCTTCC	55	150	This study
		R	CTCATCAATGATCTGGGCTTTACG	204		This study
A0KJN3	Ferrichrome-iron receptor	F	GCTTCGGTCTTCCACATCAC	1,606	176	This study
		R	ATCTCCATATCCTGACGGGTATAG	1,781		This study
R4W0J5	Ferrichrome receptor	F	GGCAAGAACGAGAAGCAGTATG	1,216	150	This study
		R	CTTGTAGGATTGGCTGGTGTTG	1,365		This study
A0KHF6	Maltoporin	F	CCTTCGCCGTTGATTTCCAC	125	165	This study
		R	TCTTGCCGTCCTTGTTGAATAC	289		This study
R4VCH3	Outer membrane porin protein	F	TACAACCAGAACGACACCAAAC	73	165	This study
		R	GTATTCAGCGAAGCCGAACG	237		This study
R4VG84	Peptide ABC transporter periplasmic peptide-binding protein	F	GACAACAAGACGGTGCTGAC	52	165	This study
		R	CAGACGATAGACGGGTTTGC	216		This study
R4VJ80	ATP synthase subunit beta	F	GGGTCTGGTGCTGGAAGTTC	117	123	This study
		R	ACCTGGATGGATTTACCGCTATTC	239		This study
R4V7Y6	Cytochrome c4	F	TCTGAACAGGACATGGAAGACC	268	198	This study
		R	GGACAGGCTCGGATACTTGG	465		This study
R4VH33	Cytochrome c551 peroxidase	F	ACTCCAGCCTCAACTTCGTG	269	181	This study
		R	AAGTCCGCCTTGCCATACAC	449		This study
A0KJP9	TonB-dependent siderophore receptor	F	AGACCGATCTGATGGATGACTC	944	168	This study
		R	CATTGGTGATGCTGCGACTG	1,111		This study
A0KL28	Cytochrome c-type protein NrfB	F	GATGCCGCCTGTACCGACTG	160	192	This study
		R	GGTGCTCTGTTTGTCCTTGAAG	351		This study
R4VF53	Cytochrome c553	F	GCCTTAGCGACGAGCAGATC	95	148	This study
		R	CCTTCGGAGTGGCACTTCTTG	242		This study
R4VNL8	Cytochrome c552	F	GAGCAGGGCAAGGTCTACAC	886	144	This study
		R	CTTGAGATCGTGAATGGCATCC	1,029		This study
A0KQV7	Cytochrome c5	F	AAGGATCTGGAAGGGATTGCG	103	112	This study
		R	CATCGTGGCAGGCGAAGCAG	214		This study
R4VS58	Fumarate reductase flavoprotein subunit	F	TGAACTTCCTCAAGCACACTCTC	1,615	150	This study
		R	AGCCATTCTTCTTAGCCTCGTC	1,764		This study
R4VNP5	Fumarate hydratase	F	ACCTGCTTCGTCAAGATCGG	217	169	This study
		R	GGGCATTGTCCTTGGTGTTC	385		This study
K1JGN0	Pyruvate-flavodoxin oxidoreductase	F	GCTCAGGGCTACTTCGTCTAC	1,360	162	This study
		R	CTCCAGCAAGTCTACCGATTCG	1,521		This study
GAP-1	Glyceraldehyde 3-phosphate dehydrogenase	F	AGAGCCTCAATGCCTATCTGC	1,102	195	[53]
		R	ACCCGAACTCGTTGTCATACC	1,306		[53]

All of the quantitative Real-time PCR reactions were carried out using SYBR® Premix Ex Taq™ II (Tli RNaseH Plus) (Takara Shuzo, Otsu, Japan). The conditions used for q-PCR were 5 s at 95 °C, 34 s at 60 °C, and then 40 cycles of 30 s at 95 °C and melt curve added. Gene expression values were determined by the ∆∆Ct method, which was normalized to the level of *GAP-1* measured in each sample and expressed relative to the value obtained in the indicated control sample [54]. All reactions were performed in a CFX96 Touch™ Deep Well Real-Time PCR Detection System (Bio-Rad). All primer sequences were designed using the software Primer Premier5. All experiments were performed at least in triplicate, independently.

### Measurement of enzyme activity

The H^+^-ATP synthase activities of *A. hydrophila* in 0, 150, 200, and 300 μM DIP were measured spectrophotometrically at 340 nm using the H^+^-ATP synthase assay kit (Genmed Scientifics Inc., MA, USA) according to the manufacturer’s protocol, which was based on the oxidation of reduced NADH to NAD^+^, by the catalysis of lactate dehydrogenase.

### In Vivo exogenous metabolite stimulation assay

The *A. hydrophila s*train was diluted at 1:100 in 300 μM DIP overnight, with or without 2, 4, and 8 mM oxaloacetic acid (OAA), α-ketoglutarate (α-KA), succinic acid (SA), and citric acid (CA) treatment at 30 °C with shaking at 200 rpm. The cell density was determined via optical density (OD) measurement at 600 nm.

### H^+^-ATP synthase inhibition assay

For the H^+^-ATP synthase inhibition assay, an orthogonal test was further performed. The *A. hydrophila* strain was diluted at 1:100 in fresh LB medium with a series of DIP overnight from 0, 150, 200 to 300 μM and DCCD from 5, 10, to 20 μg/mL, respectively. After incubation at 30 °C for additional 12 h, cells were diluted appropriately with fresh LB medium for bacterial colony counting, which displayed as color grading according to their survival colony accounting with the control normalized to 100 [55].

### Statistical analysis

All Statistical analysis was performed by using One-way ANOVA measurement to compare the different treatments with the control sample.

## Abbreviations

0.2 M Tris–HCl, pH = 8.0, 0.5 M sucrose, 1 mM Na_2_EDTA, TSE buffer; ABC, ATP-binding cassette; BLAST, Basic local alignment search tool; CA, Citric acid; DCCD, N, N’-Dicyclohexylcarbodiimide; DIP, 2, 2′-dipyridyl; FDR, False discovery rate; GAP-1, Glyceraldehyde-3-phosphate dehydrogenase; GO, Gene Ontology; HCD, High-energy collision dissociation; IAA, Iodoacetamide; IMPs, Inner membrane proteins; LB, Luria- Bertani; LC MS/MS, Liquid chromatography tandem mass spectrometry; OAA, Oxaloacetic acid; OD, Optical density; OMPs, Outer membrane proteins; PMF, Proton motive force; q-PCR, Quantitative polymerase chain reaction; ROS, Reactive oxygen species; SA, Succinic acid; TCA, Tricarboxylic acid; TCEP, Tris (2-carboxyethyl) phosphine; TEAB, Triethylammonium bicarbonate; α-KA, α-Ketoglutarate.

## Additional files

Additional file 1: Table S1. Identification and quantification results using dimethyl labeling based quantitative proteomics. (XLSX 834 kb) Additional file 2: Figure S1. Schematic representation of siderophore-mediated iron uptake systems and the influence of iron depletion on the cell envelope in *A. hydrophila*, according to quantitative proteomic analysis. (TIF 2163 kb)
