# Supplementary figures and images for: Quantitative proteomic analysis of cell envelope preparations under iron starvation stress in Aeromonas hydrophila
Source: BMC Microbiol. 2016 Jul 22;16:161. doi: 10.1186/s12866-016-0769-5 (PMC4957856; doi:10.1186/s12866-016-0769-5)

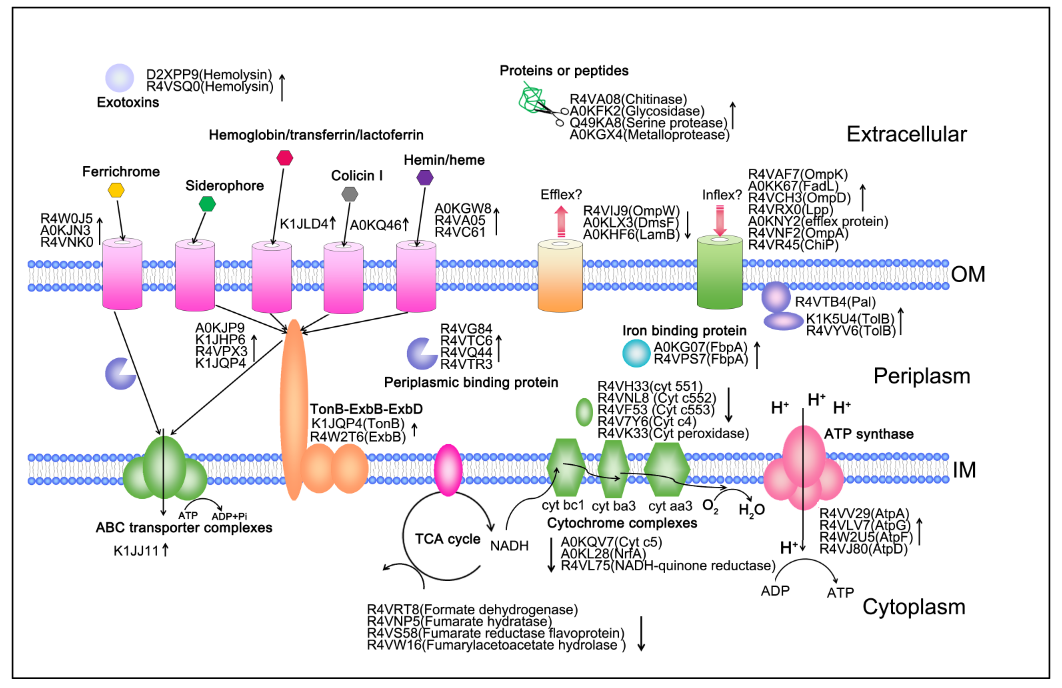

Supplement: Additional file 2: Figure S1. — Schematic representation of siderophore-mediated iron uptake systems and the influence of iron depletion on the cell envelope in A. hydrophila, according to quantitative proteomic analysis. (TIF 2163 kb) [file 12866_2016_769_MOESM2_ESM.tif]
